# The potential of gas plasma technology for targeting breast cancer

**DOI:** 10.1002/ctm2.1022

**Published:** 2022-08-22

**Authors:** Sander Bekeschus, Fariba Saadati, Steffen Emmert

**Affiliations:** ^1^ ZIK plasmatis Leibniz Institute for Plasma Science and Technology (INP) Greifswald Germany; ^2^ Clinic and Policlinic for Dermatology and Venereology Rostock University Medical Center Rostock Germany

**Keywords:** adenocarcinoma, adjuvant therapy, ICD, immunogenic cell death, palliation, plasma medicine, reactive oxygen species, ROS

## Abstract

Despite therapeutic improvements in recent years, breast cancer remains an often fatal disease. In addition, breast cancer ulceration may occur during late stages, further complicating therapeutic or palliative interventions. In the past decade, a novel technology received significant attention in the medical field: gas plasma. This topical treatment relies on the partial ionization of gases that simultaneously produce a plethora of reactive oxygen and nitrogen species (ROS/RNS). Such local ROS/RNS overload inactivates tumour cells in a non‐necrotic manner and was recently identified to induce immunogenic cancer cell death (ICD). ICD promotes dendritic cell maturation and amplifies antitumour immunity capable of targeting breast cancer metastases. Gas plasma technology was also shown to provide additive toxicity in combination with radio and chemotherapy and re‐sensitized drug‐resistant breast cancer cells. This work outlines the assets of gas plasma technology as a novel tool for targeting breast cancer by summarizing the action of plasma devices, the roles of ROS, signalling pathways, modes of cell death, combination therapies and immunological consequences of gas plasma exposure in breast cancer cells in vitro, in vivo, and in patient‐derived microtissues ex vivo.

## Graphical abstract

- Gas plasma is a multi‐ROS/RNS generating technology with potent antitumour activity.
- Gas plasma exposure elicits immunogenic cancer cell death (ICD) and provides immunostimulation against breast cancer in vivo.
- Gas plasma technology has considerable potential to be used as novel neoadjuvant or adjuvant physical treatment modality for targeting ulcerating breast cancer within in situ vaccination schemes.

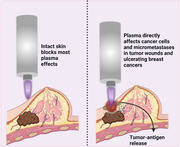

## 1 INTRODUCTION

Breast cancer is the most common cancer diagnosed in women worldwide. It is assumed that breast cancer cells derive from epithelial tissue of the breast,^1^ while bipotential (mammary epithelial) stem cells are also discussed as tumour‐initiating events.^2^ Approved therapies such as surgery, chemotherapy, radiotherapy, hormone therapy and immunotherapy have contributed significantly to breast cancer treatment and increased the patients’ overall survival. The use of a specific breast cancer therapy depends on the origin, stage and other breast tumour characteristics. Surgery is the most common type of treatment due to the lack of systematic side effects and complications. In addition, other therapeutic techniques are used to prevent the recurrence of the disease.^3^ However, the resistance of tumour cells to induced programmed cell death by such treatments and the ability to invade host tissues and metastasize to distant sites is one of the most critical challenges in this disease. The annual number of people dying from breast cancer and suffering from various therapeutic side effects shows that more research is needed to find alternative or complementary therapies.^4^, ^5^ Ulcerating breast tumours occur when tumour cells under the skin break through the skin surface and appear as an ulcer in various areas such as the primary tumour site or metastasis site such as lymph nodes. These ulcers can become infected due to various factors, which causes severe problems for patients. Symptoms of these ulcers include foul odor, unpleasant discharge, pain, bleeding and itching.^6^

Cold physical plasma, an ionized gas generated near room temperature, demonstrated impressive capabilities in cancer therapy.^7^ Many pre‐clinical models have been employed to evaluate the efficacy of gas plasma on different types of cancers. The central concept in the field of plasma medicine is the generation of reactive oxygen and nitrogen species (collectively referred to as reactive oxygen species (ROS), as and reactive nitrogen species (RNS) also contain reactive oxygen in most cases) by gas plasma, referred to hereafter as ‘plasma’. Extensive reactivity, damage to cells and tissue at higher concentrations, and generation of new ROS by chain reactions are the known characteristics of ROS. ROS are involved in cellular regulation and signalling at low concentrations, while at higher concentrations, their effect can be deleterious^8^ but simultaneously also therapeutic in the context of, for example, cancer.^9^ Accordingly, gas plasma technology is complementary to existing local physical treatment modalities that were shown to partially or fully rely on the generation of high ROS levels, such as photodynamic therapy,^10^ radiotherapy,^11^ electrochemotherapy^12^ and hyperthermia.^13^ Endogenously generated ROS and oxidative stress in the breast cancer tumour microenvironment (TME) have received increasing attention recently,^14^ and multi‐species gas plasma ROS applications may redox modulate the TME potentially in favor of breast cancer therapy, which is the focus of this review, complementing previous summaries on the prospects of gas plasma treatment in, for example, melanoma,^15^ glioblastoma,^16^ osteosarcoma^17^ and pancreatic^18^ and head and neck cancer.^19^

### 1.1 Breast cancer

**FIGURE 1:**
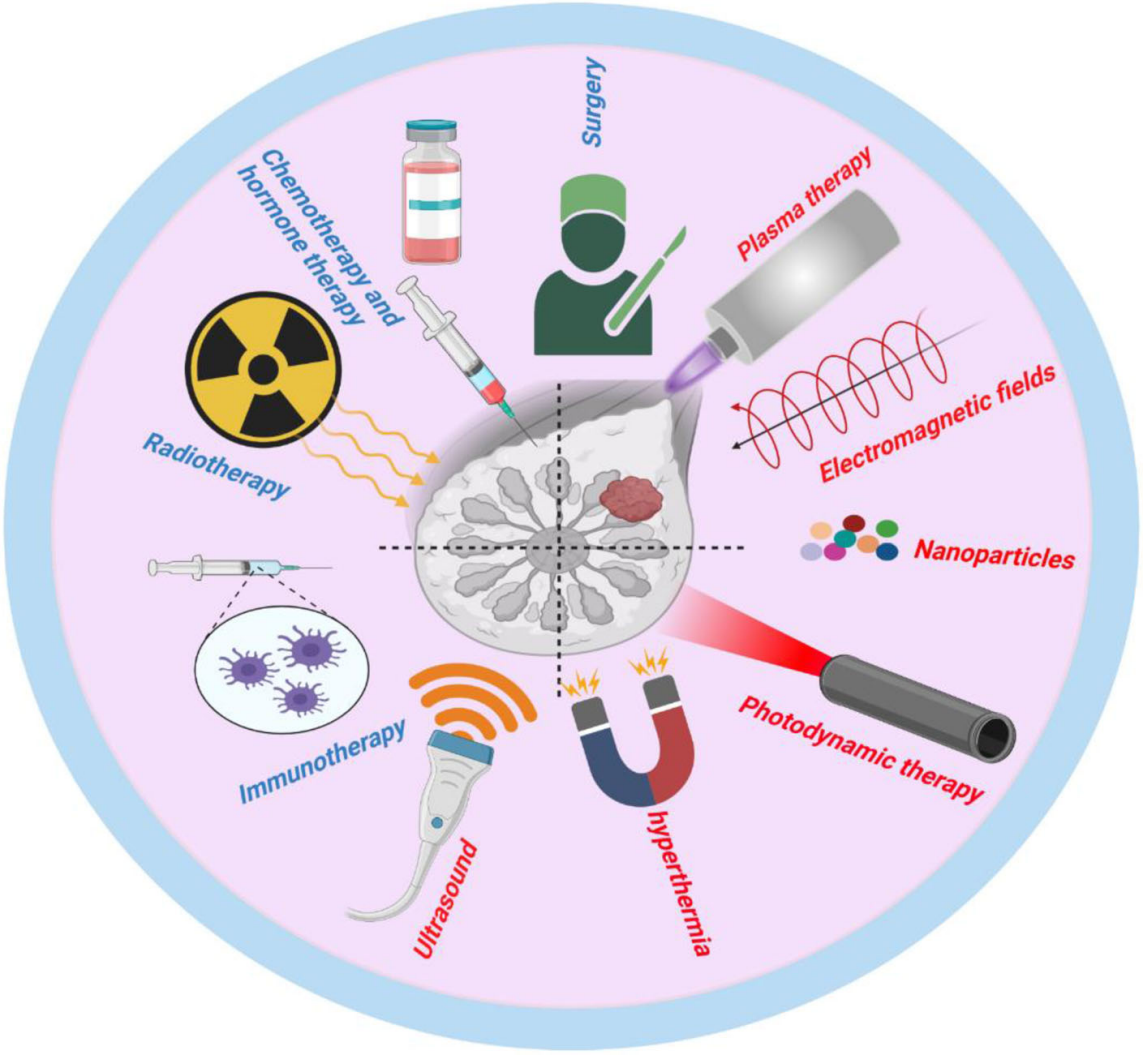
Currently approved (blue) and experimental (red) treatment modalities for breast cancer treatment

Breast cancer is the most common and second leading cause of cancer death in women. In 2020, 2.3 million women were diagnosed with breast cancer, and 685 000 breast cancer deaths were recorded globally.^20^ These numbers are striking and motivate a better understanding of breast cancer and its existing and potential therapies. Breast cancer is a complex and multifactorial disease affected by genetic and environmental factors. Breast cancer is generally divided into two categories based on the site from which the tumour originated. Lobular breast cancer instigates in the breast's milk‐producing glands, while ductal breast cancer presents in the gland's ducts.^21^ Several risk factors are associated with breast cancer, such as family history, aging, early menstruation, late menopause, overuse of alcohol and a high‐fat diet.^22^ On the molecular levels, epigenetics (e.g., altered DNA methylation and histone modifications) and mutations of oncogenes and tumour suppressor genes such as BRCA1, BRCA2, P53, RB1, PTEN, NME1, CCND1, PIK3CA, CDH1, ATM, FHIT and Mapsin can trigger this disease.^23^, ^24^ Different kinds of systemic and non‐systemic treatment are selected for breast cancer therapy based on the tumour's molecular subtype, stage and location. Surgery, radiotherapy, chemotherapy, hormone therapy, immunotherapy and targeted therapy alone or in combination are the most common breast cancer treatment methods (Figure 1). Classical multimodal breast cancer treatment schemes include surgery for tumour removal or mastectomy followed by chemotherapy or radiotherapy.^25^ The adjuvant or neoadjuvant chemotherapy is often comprised of taxanes and anthracyclines, together with adjuvant hormonal suppression treatments (e.g., tamoxifen) in the case of progesterone or estrogen receptor positivity.^26^ For radiotherapy, there are different modalities recommended in curative or palliative treatment intentions, and specific details of target tissue exposure (e.g., ductal carcinoma in situ, accelerated partial breast irradiation, and regional nodal irradiation) and timing are still a matter of active research.^27^ Compared to other types of cancers, the 5‐year survival is relatively high but nevertheless dramatically impacts the overall number of cases because of the high prevalence of breast cancer.

Many therapies come with various adverse and severe side effects such as organ damage and drastically reduced overall quality of life.^28^ For instance, rehabilitation needs already in early‐stage breast cancer patients include focal problems due to local therapies (e.g., pain, lymphedema, arm and should problems), systemic issues related to treatment (e.g., neuropathy and fertility), and psychosocial illness (e.g., depression, sleep disturbances, and fatigue) that require attention.^29^ Disease recurrence is a significant issue after surgical excision of the primary tumour that is propelled by tumour micrometastases and breast cancer stem cells.^30^ In case of evident or suspected tumour metastases, the main goal of chemotherapy is to eradicate the breast cancer microcolonies systemically and loco‐regionally via, for instance, interference in cell cycle progression.^31^ Unfortunately, intrinsic or acquired resistance of breast cancer cells to chemotherapy, especially in the aggressive triple‐negative breast cancer (TNBC) molecular type, hinders therapeutic success in many cases.^32^ Patients undergoing radiotherapy suffer from irreversible skin damage and significant weight loss following non‐malignant cell damage.^33^ Hormone therapy imposes high medical costs for patients and prolongs treatment, which can promote invasive growth of malignant tumours.^34^ Altogether, tumour immune evasion, heterogeneity, resistance to treatment and apoptosis, invasion and metastasis are hallmarks of tumour progression that enable tumour growth and promote treatment failure, causing disease fatality.^35^ Hence, there is a constant need to investigate novel research lines in cancer therapy in general and breast cancer in particular. This includes not only systemic treatments but also local therapy modalities that might be adjuvant or neoadjuvant to approved and first‐line treatments.

### 1.2 Gas plasma technology

**FIGURE 2:**
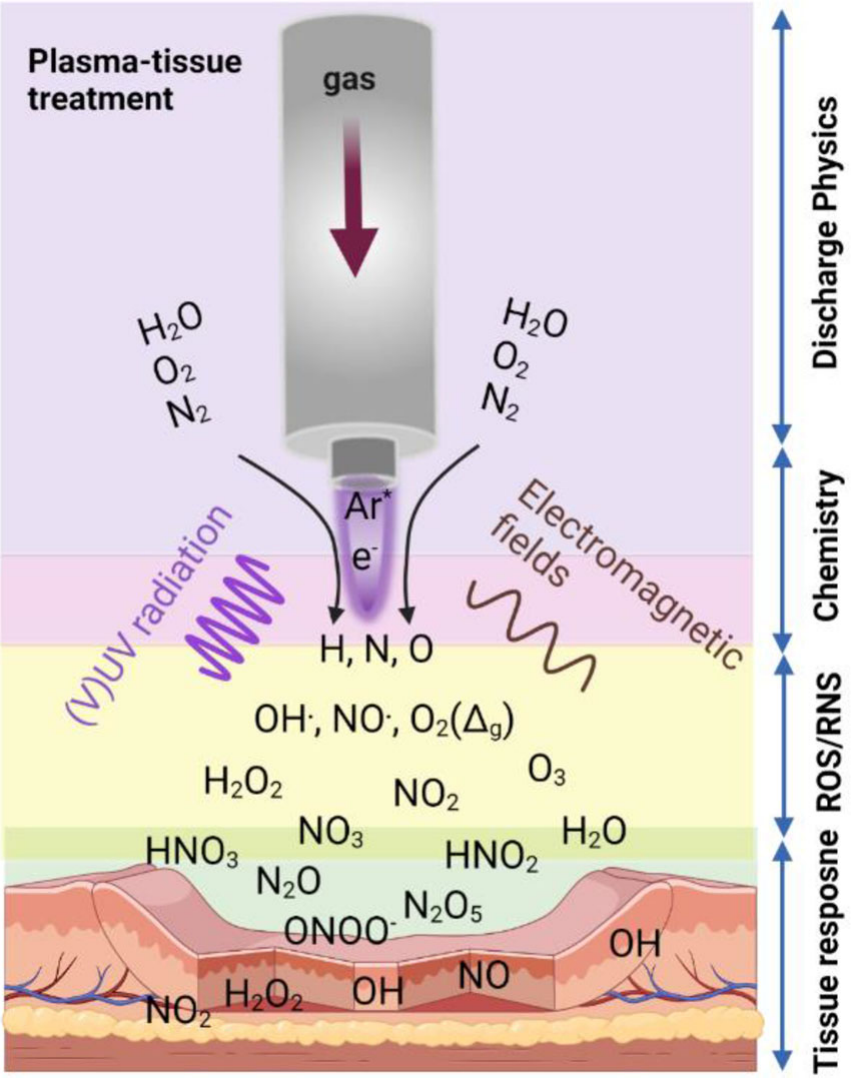
Schematic of gas plasma tissue treatment and potential mediators and effectors

Physical plasma is described as the so‐called fourth state of matter. These plasmas are generated by partial ionization of a gas, usually a noble gas in the case of plasma jets for medical applications. The partially ionized gases exhibit different chemical and physical properties, including the generation of ROS and RNS when in contact with ambient air's oxygen and nitrogen, low‐dose UV radiation, visible light, electromagnetic fields and electrons and ions (Figure 2). The leap innovation in using this technology was the production of so‐called cold physical plasmas at atmospheric pressures. Before, plasmas were easily ignited in many technical processes, such as halogen lamps; however, usually under lower‐than‐atmospheric pressure. At the same time, hotter plasmas are known from other technical processes, such as welding and discharges during switching. Generating a highly reactive gas at about room temperature enabled several putative applications, including the treatment and functionalization of heat‐sensitive materials and surfaces as well as medical treatments. This was the birth hour of the research field of plasma medicine.^36^

**FIGURE 3:**
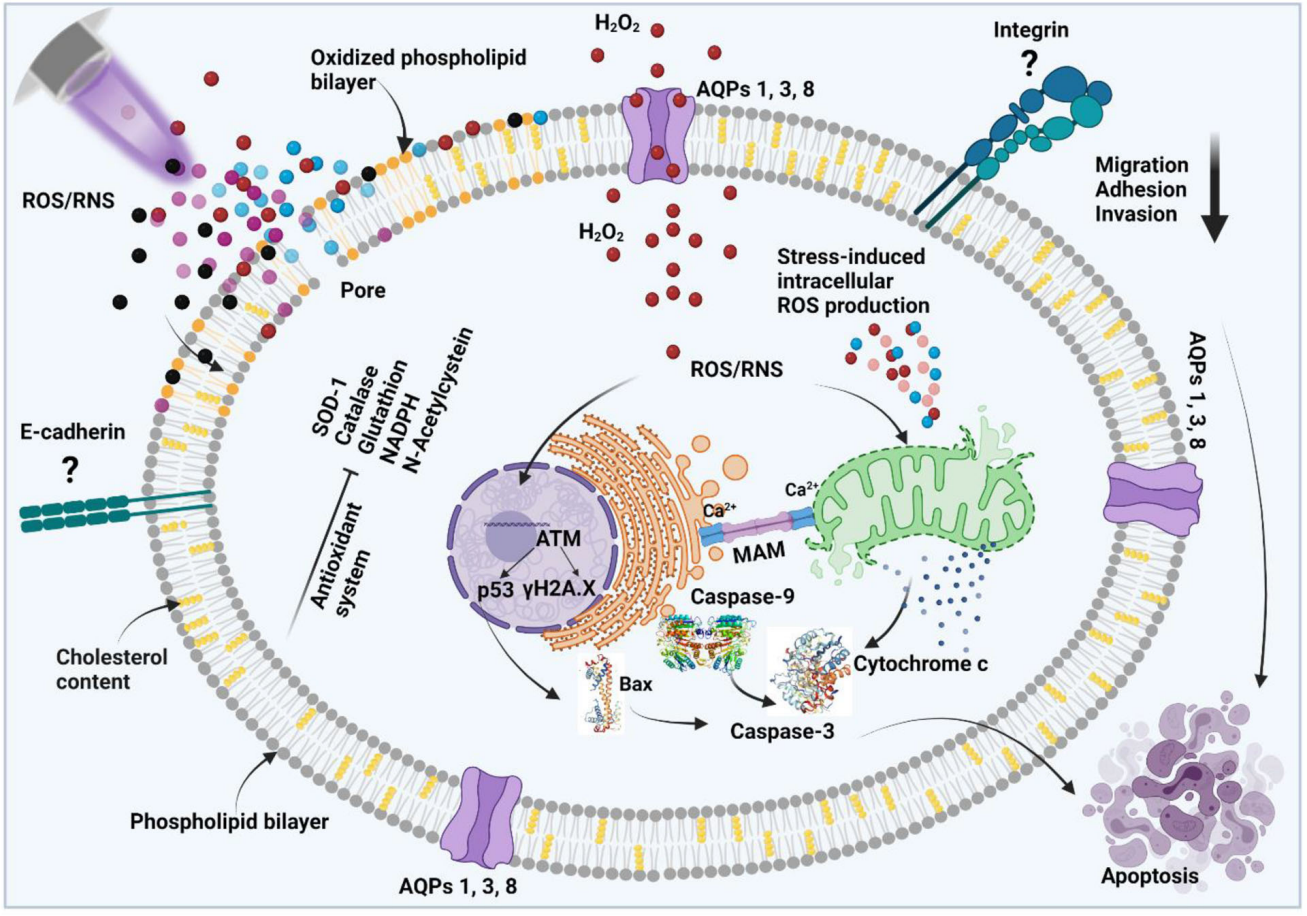
Illustration shows selected effects proven or hypothesized to be important in gas plasma‐mediated breast cancer cell demise

Today, it is consent that the majority of biomedical plasma effects observed are based on the plasma sources’ ROS/RNS generation.^37^ At the same time, several studies revealed that the effects of other physical plasma components, such as low‐dose UV‐radiation and electric fields, especially in terms of cellular toxicity, are negligible. This is especially true for in vitro studies where excess liquid surrounds the treated target in question, such as microorganisms and eukaryotic cells in 2D monolayers. It is also clear that in those situations, the effect of the long‐lived oxidants—chemical reaction products of the short‐lived ROS/RNS produced by plasma—dominates the biomedical effects observed. In the absence of excessive amounts of water, such as the clinical application of dry but infected skin,^38^ short‐lived molecules will directly oxidize proteins and lipids without deteriorating to less reactive but more long‐lived species as in liquids. In the case of wet tissues or wounds without protecting keratin layers, both short‐ and long‐lived species will probably be generated and effective. A unifying model for in situ redox chemistry of plasma‐treated tissues does not exist. This is because the experimental proof and unambiguous identification of ROS/RNS in tissues, potentially spatiotemporally resolved, is technically not possible until today.^39^ The lack of information on ROS types and concentrations in tissues hampers the understanding of ROS‐generating treatments, including certain chemotherapeutics,^9^ as well as pinpointing clear roles of ROS in the pathophysiology of the TME in breast cancer). Major potential mechanisms of gas plasma effects in cells have been summarized in Figure 3.

Currently, only a handful of devices have been approved based on clinical results and thorough scientific investigations in the EU as medical device class IIa for application in dermatology.^40^ Clinical plasma applications can be divided into experimental and guideline‐directed applications. Plasmas have potent antimicrobial effects evident in vitro and in wounds in vivo^41^, ^42^ and support the healing of chronic wounds and ulcers. The appropriate use of gas plasma technology in medicine has been implemented in an S2k medical guideline by the Association of the Scientific Medical Societies in Germany for the first time worldwide in early 2022. The consent document was produced by over 10 medical societies, such as the German Societies for Dermatology, Periodontology, Ophthalmology, General and Visceral Surgery, and Head and Neck Surgery. At the same time, clinically approved plasma technology is currently observed by German institutions responsible for health insurance reimbursements. Interestingly, a recent randomized clinical trial indicated improved wound healing with plasma jet application independent of antimicrobial effects.^43^ Therefore, plasma technology has solidly entered daily medical practice for some indications. Experimental clinical plasma applications include several dermatology diseases, such as fungal and viral skin infections and actinic keratosis (carcinoma in situ) treatment.^44^, ^45^ Antiviral effects of gas plasma have been described for long and re‐gained importance during the COVID19‐pandemic in the form of room air and water disinfection devices and antiviral plasma treatment of abiotic and biotic surfaces.^46^, ^47^ In addition, plasma cancer applications have already been suggested for more than 15 years. Often, the question arises how these two seemingly distinct processes, that is, healing and toxic action, could be achieved with the same method. The answer is hormesis, a well‐described phenome in pharmacology describing opposite effects of the same compound in the host, depending on its low or high concentration.^48^ It has been known in the field of redox biology and medicine for long that ROS and RNS perform hormetic actions. At low concentrations, hydrogen peroxide acts as a signalling molecule, intertwined in redox signalling cascades, even providing growth stimulation under certain circumstances. However, redox signalling is disrupted at higher concentrations, and toxic effects dominate.^49^ Another example is nitric oxide (NO), which stimulates vasodilation at lower concentrations, and is intentionally produced by macrophages at higher concentrations for antimicrobial defense purposes. Since gas plasma devices produce ROS, and ROS are the biochemical mediator of biomedical gas plasma exposure effects, the hormesis concept of ROS also applies to this technology, inevitably making the field of plasma medicine a part of the redox biology and medicine field: applied redox medicine.^50^ Accordingly, short plasma treatment times or energies have stimulating properties beneficial in, for example, defective wound healing, while extended treatment times or higher energies provide tumour control.^37^

Its superior safety profile is a prime hallmark of gas plasma technology in medicine. Several clinical studies have concluded a lack of severe adverse events or notable side effects.^41^, ^43^, ^51^, ^52^, ^53^, ^54^, ^55^, ^56^, ^57^, ^58^, ^59^ Besides occasional mild stitching and modest ozone generation, gas plasma exposure is exceptionally well tolerated. No local anesthesia is required. Two long‐term follow‐up studies of 1‐year and 5‐years demonstrated an absence of abnormal effects in gas plasma‐treated tissues.^60^, ^61^ Similar conclusions were reached in two long‐term in vivo studies. One is on gas plasma‐treated ear wounds and animal analysis by pathology, PET‐CT, MRI and molecular analysis 1 year later, reporting an absence of abnormal development or tumour formation in more than 80 animals.^62^ The second study included over 400 animals exposed to gas plasma alone or in combination with the carcinogenic agent DBP in the oral mucosa monthly for 1 year. Gas plasma did not lead to negative long‐term effects. It did not promote the pro‐carcinogenic action of DBP exposure to accelerate lesion or invasive tumour formation, as confirmed by thorough macroscopic and pathology investigations as well as molecular profiling of 140 transcripts.^63^ It should be noted that 1 year in mice translates to about 60 years of life in humans. On the molecular level, several studies were performed based on OECD protocols to analyze the genotoxic potential of gas plasma exposure of cells. Several studies found no genotoxic effects for different types of gas plasmas devices.^64^, ^65^, ^66^, ^67^, ^68^, ^69^ Many studies,^70^, ^71^ including our own,^72^, ^73^ reported extensive histone 2A.X phosphorylation following gas plasma exposure, which is a biomarker for double‐strand breaks in radio‐biology. However, the effects observed with gas plasma exposure are related to the pleiotropic roles of this molecule, namely in response to oxidative stress, apoptosis and DNA‐damage response initiation, that in turn is also intertwined with oxidative stress. Therefore, γH2A.X is not a suitable biomarker for genotoxic events in gas plasma‐treated cells and tissues.^74^

### 1.3 ROS

ROS are small, unstable, highly reactive molecules formed by electron addition or subtraction of oxygen to gain stability due to unpaired electrons in its orbitals. The ROS abbreviation sometimes also covers RNS, as the latter often contain reactive oxygen. Upon contact with biomolecules, such as lipids, proteins and nucleic acids, ROS mostly oxidize these by transferring electrons.^75^ The body is frequently exposed to exogenous ROS from environmental stressors, such as UV radiation, smoking and air pollution. In addition to exogenous factors, endogenous sources like mitochondrial, microsomal and peroxisomal activity in the electron transport chain and exacerbated activity of ROS/RNS‐generating enzymes in phagocytes, such as NOX and MPO, can elevate oxidative stress locally.^76^ Simultaneously, cells have various enzymatic and non‐enzymatic antioxidants to detoxify ROS, such as glutathione, thioredoxins, superoxide dismutases, catalase and peroxidases.^77^ It is well known that excessive and/or chronic oxidative stress can induce redox status imbalance, lipid peroxidation and ultimately apoptosis.^78^ Several chemotherapeutics, such as anthracyclines, are related to oxidative stress induction.^79^ ROS production is also the basis of photodynamic therapy^80^ and part of the action of radiotherapy, also in breast cancer.^81^ Importantly, oxidative stress can also be combined with existing oncological therapies. Ogawa and colleagues reported that applying H_2_O_2_ before radiotherapy renders cancer cells more sensitive to radiotherapy.^82^

Gas plasma‐induced ROS/RNS can increase intracellular reactive species levels and decrease the antioxidant capacity of the target cells.^83^ Common ROS/RNS produced by gas plasmas are hydroxyl radicals, atomic oxygen, superoxide radical, NO, peroxynitrite, singlet delta oxygen, ozone and others. The ROS/RNS redox chemistry in the plasma gas phase is highly dynamic and also depends on the plasma device in question, with hundreds of different reactions taking place in a concise time frame.^84^, ^85^ The species being deposited in liquid matrices are less plentiful but still more than a dozen.^86^ The plasma‐derived ROS can be delivered via two modalities, direct and indirect plasma treatment. Direct plasma treatment refers to the plasma source producing a gas plasma that is in direct contact with or slightly above the treatment target to unleash its active cocktail of components for therapeutic purposes. This treatment contains all plasma effectors, including ROS, and has the most potent activity per treatment time.^87^ By contrast, indirect treatment involves plasma treatment of a liquid that can be used for treatment immediately or stored for later use. A promising application is the combination with existing chemotherapy in the approved hyperthermal intraperitoneal chemotherapy or pressurized intraperitoneal aerosol chemotherapy for targeting peritoneal carcinomatosis or for treating bone cancer.^88^ These liquids, however, mainly contain long‐lived species that could also be generated independently of plasma processes.

Plasma‐derived ROS for therapy can be produced by basically two categories of plasma devices. Plasma jets require a gas, usually a noble gas (due to its easy excitation), but ambient or pressurized air is also used in some devices, actively fed into the plasma zone.^89^ There, the gas becomes partially ionized (reactive) and is expelled into the ambient air, where chemical processes produce reactive oxygen and nitrogen species from ambient oxygen and nitrogen. The advantage of plasma jets is the scalpel‐like application in medical practice, helping the health care staff visually track the target's treatment. Also, plasma jets are very focused and efficiently enter even the smallest cavities, such as hair follicles and tooth‐root channels.^90^, ^91^ Yet, their treatment area is limited. By contrast, dielectric barrier discharges can cover larger areas to treat simultaneously.^92^ However, their geometry is flat, leading to uneven treatment of non‐flat surfaces as frequently the case in many medical fields. Moreover, the plasma treatment of the target cannot be tracked visually, as the device covers the area on which it expels its active cocktail. So‐called plasma multi‐jets, devices with multiple single plasma jets combined to cover larger areas, may combine the benefits of both systems but have only started to be explored.^93^, ^94^, ^95^ A frequent debate is whether plasma therapy can be ‘dosed’. Unfortunately, there is no consensus yet on how such dose would be defined in such a multi‐component and multi‐ROS technology with less clear individual contributions of each component and supposedly different therapeutic needs across several diseases.

A future dosing concept based on ROS generation is likely, however. By contrast, in radiotherapy, dosing is already a long‐standing concept measured in Gray (Gy). The treatment modality is mediated by either electromagnetic (e.g., X‐ and gamma rays) or particulate radiation (electrons, protons, neutrons). The biological principle of this ionizing radiation in the treated target tissue is the dislodgment of electrons from molecules. Ultimately, this produces free radicals in all cellular microenvironments,^96^ including extracellular, intracellular, intravesicular and intranuclear compartments. This starkly contrasts with gas plasma technology, where ionization occurs in the active plasma zone but not in the target tissue since the electromagnetic energies in plasmas are too weak. Yet, the similarity with both technologies is ROS generation, with gas short‐lived plasma‐derived ROS only reaching extracellular targets and cell membranes at sufficient concentrations, as they are transported to the cells and not generated within the cells as the case with radiotherapy. This explains the perhaps greatest difference between both modalities, the penetration depths, which is not an obstacle in radiotherapy. On the contrary, gas plasmas only act topically on the target tissue within the first dozen micrometers to confer primary effects,^97^, ^98^ while secondary effects can occur up to several millimeters in the tissue.^99^, ^100^ Radiotherapy is usually applied in fractionated regimens; breast cancer frequently receives hypofractionation.^101^ Also gas plasma therapy, within its current indications (e.g., chronic wound healing^40^), is applied several times. By definition, this is not necessarily fractionation, as a total therapeutic dose for the disease or target‐ot‐treat is not definable (yet). Usually, gas plasma treatment is endorsed as long as there is an objective therapeutic benefit. This was also the case for the small cohort of palliative head and neck cancer patients receiving a few to several dozen gas plasma treatment cycles in the clinical setting, with partially remarkable results, before tumour progression continued.^102^, ^103^ Similarities and differences between radiotherapy and gas plasma technology are briefly summarized below (Table 1).

**TABLE 1:** Brief comparison between radiotherapy and gas plasma technology

**Category**	**Radiotherapy**	**Gas plasma therapy**
Mode of action	Unimodal; ionizing radiation unleashes electrons and generates ions, producing reactive species and direct defects on biomolecules	Multimodal; gas plasma ionization generates several components, such as electric fields, ions, electrons, ultraviolet and visible light, and dozens of reactive oxygen and nitrogen species simultaneously
Potentially mutagenic	Yes	No
Penetration depth	High, not a limitation in clinical application	Low, only surface or near‐surface primary (a few dozen micrometres) or secondary (millimetres) effects
Cancer therapy approval	Yes	No, but approved if tumour wound antisepsis is the goal, e.g., ulcerating and infected breast and head and neck cancer wounds
Fractionation and dose	Yes, usually the case based on dose	Yes, usually therapeutic effects in clinics always require multiple treatment cycles that are however continued based on objective responses and not on overall dose; no overall dosing concept available yet
Effectors	Radiations effects all biomolecules, regardless of their localization being intracellular or extracellular	Extracellular ROS/RNS generation and delivery to cells, only membrane oxidation or passive ROS/RNS delivery to intracellular sites
Electron energies	Very high (>50 000 electron volts)	Very low (∼1–10 electron volts for clinical plasma devices)

Abbreviation: ROS/RNS, reactive oxygen and nitrogen species.

### 1.4 Tumour immunology and immunogenic cancer cell death

According to McFarlane's immune surveillance theory, the immune system can recognize and destroy tumour cells.^104^ In brief, tumours can stimulate their own demise by stimulating anticancer immunity via the expression of tumour‐associated antigens or neoantigens considered foreign to the immune system.^105^ The efficiency of this process is also related to the tumour's mutational burden, which differs considerably between cancer types.^106^ At the same time, the lack of expression of immunogenic antigens, decreased expression of MHC class I molecules, inadequate stimulation of T lymphocytes, production of immunosuppressive or immune cell‐killing factors, and immunosuppressive cells such as regulatory T‐ cells are among the most critical mechanisms of tumour escape from immune responses. This is because immune‐mediated tumour cells removal involuntarily puts evolutionary pressure on tumour cells to mutate further and generate variants efficient in evading immune responses by either down‐regulating pro‐immunogenic features (such as expression of HLA molecules) or up‐regulating immunosuppressive factors, for example, immune checkpoint receptors and ligands, such as PD‐L1 and CTLA4. The discovery of the therapeutic blockade of immune checkpoints via antibodies was acknowledged with the Nobel Prize in Physiology or Medicine in 2018.^107^ Inhibitory immune receptors, which have been identified in breast cancer, are PD‐1, CTL‐4, PD‐L1, TIM3, TIGIT, BTLA and LAG3.^108^ The most common immune checkpoint antibodies approved for breast cancer treatment are atezolizumab, pembrolizumab and dostarlimab, which target the PD‐1/PD‐L1 pathway^109^, ^110^ (Table 2). Clinicians focused on combining standard of care treatment with immunotherapy to target breast cancer more efficiently. The general concepts of empowering antitumour immunity have been reviewed thoroughly before.^111^, ^112^ Several cell types participate in this process, and especially cytotoxic T‐cells are viewed as the most efficient effectors targeting cancer cells.^113^, ^114^

**TABLE 2:** FDA‐approved checkpoint antibody immunotherapies for breast cancer

**Drug name**	**Target protein**	**Mechanism of action**
Atezolizumab (Tecentriq)	PD‐L1	Humanized IgG1κ monoclonal antibody against PD‐L1 expressed on tumour and non‐tumour cells; inhibits the binding of PD‐L1 to PD‐1 predominantly expressed on T‐cells and thereby blocking a major immunosuppressive pathway.
Pembrolizumab (Keytruda)	PD‐1	Humanized IgG4 monoclonal antibody against PD‐1 expressed predominantly on T‐cells; inhibits the binding of PD‐L1 expressed on tumour and non‐tumour cells to PD‐1 and thereby blocking a major immunosuppressive pathway.
Dostarlimab (Jemperli)	PD‐1	Humanized IgG4 monoclonal antibody against PD‐1 expressed predominantly on T‐cells; inhibits the binding of PD‐L1 expressed on tumour and non‐tumour cells to PD‐1 and thereby blocking a major immunosuppressive pathway; approved to treat mismatch repair deficient (dMMR) advanced‐stage breast cancer that had emerged during or after therapy.

One bottleneck of generating antitumour T‐cells is the supply of tumour antigens in a pro‐inflammatory fashion. The concept of immunogenic cancer cell death (ICD) predicts this form of sterile and pro‐inflammatory cell death to promote the generation of T‐cells targeting cancer. The concept has been reviewed several times.^115^, ^116^ The idea is that a given agent (e.g., anthracyclines chemotherapy) or process (e.g., photodynamic therapy or radiotherapy) promotes local tumour cell death concomitant with the release of damage‐associated molecular patterns (DAMPs), such as ATP, HSP70 and HMGB1,^117^ together with endoplasmic reticulum stress that promotes externalization of eat‐me signals, such as calreticulin (CRT).^118^ Dendritic cells (DCs) in TME phagocytose the tumour material, and the DAMPs lead to their maturation and activation. Subsequently, the DCs migrate to sentinel (draining) lymph nodes where they (cross‐) present tumour antigen to T‐cells. Insufficient co‐stimulation during this process can lead to T‐cell anergy or failure of T‐cell activation. Since DCs have phagocytosed the tumour antigen in a pro‐inflammatory (ICD) environment in the context of DAMPs, antigen presentation to T‐cells is above threshold and cognate T‐cells proliferate. The clonal expansion provides the body with systemic anticancer immunity, also targeting metastases not treated initially. It is later described that gas plasma breast cancer treatment was capable of eliciting such a cancer‐immunity‐cycle.^119^ This concept is generally attractive for local therapies and can be combined with checkpoint immunotherapy to spur further the quality or quantity of the newly generated T‐cells’ activity.

## 2 PLASMA BREAST CANCER TREATMENT AND MECHANISM OF ACTION

Many studies have investigated the effects of gas plasma treatment on breast cancer cells in vitro (Table 3) and in vivo (Table 4); clinical gas plasma breast cancer treatments have not been reported so far. The reports addressed various questions, ranging from cell death modalities, over combination treatments with existing cancer treatment regimens, to drug re‐sensitization and immunological consequences. Moreover, our recent studies investigated for the first time gas plasma toxicity in patient‐derived breast cancer tissues exposed ex vivo.^120^ Markedly, overall similar effects were noted across most studies, independent of the design and engineering of the plasma device used, and dependent on ROS generation of the plasma device. The majority of studies used the cell lines MDA‐MD‐231 and MCF‐7, apart from about a dozen other cell lines utilized to a lesser extent. MCF‐10A was often referenced as normal (non‐cancerous) breast cell line. All treatments showed overall dose‐ or treatment time‐dependent effects regarding gas plasma‐derived ROS production and cytotoxic or growth‐inhibiting effects in the breast cancer cell lines.

**TABLE 3:** In vitro studies and findings in gas plasma breast cancer treatment. BC = breast cancer

Cell line (s)	Gas plasma device	Exposure modality	Gas plasma treatment effects and mechanisms of action in breast cancer cells	Ref.
MDA‐MD‐231	Plasma jet (He) direct treatment	5–120 s, 24 h and 48 h incubation	▪ Gas plasma treatment time‐dependent increase of superoxide, H_2_O_2,_ OH, NO_2_^–^, and NO_3_^–^ in treated cell culture medium ▪ Treatment‐time dependent toxicity greatest at 48 h	^121^
MCF‐7, MJ1, MN3, HBL	DBD (air) direct treatment	5–15 s, 72 h incubation	▪ The three BC cell lines demonstrated different responses in a dose‐dependent manner via caspase 9‐induced apoptosis, while gas plasma did not show toxic effect in HBL non‐malignant cells. ▪ p53 played no significant role in the apoptosis pathways	^163^
MCF‐7, T‐47D, SK‐BR‐3, BT‐474, MDA‐MB‐231, Hs578T, HCC1806	Plasma jet (He) direct treatment	120–360 s, 48 h incubation	▪ Seven BC cell lines with differing molecular profiles were analyzed via MTT assay 48 h after gas plasma exposure ▪ ER+/PR+/HER2+ cells were the most resistant to gas plasma treatment ▪ ER+/PR+/HER2+ cells were most sensitive to gas plasma treatment	^122^
MCF‐7, T‐47D, ZR‐75‐1, BT‐549, MDA‐MB‐231, Hs578T, HCC1569, MDA‐MB‐157, MDA‐MB‐175VII, HCC1954, MDA‐MB‐361, HCC1428, MDA‐MB‐468, AU‐565	Plasma jet (He or He/O_2_) direct treatment	Five different modes compared with treatment times from 10–240 s; 144 h incubation	▪ Additive toxicity of gas plasma and radiotherapy observed in the cell lines tested ▪ Cell lines sensitive to gas plasma exposure were also sensitive to radiotherapy (high correlation). ▪ The addition of O_2_ into the plasma gas inlet increased the cytotoxicity of gas plasma ▪ Gas plasma in combination with the drug olaparib has higher toxicity, hence DNA repair inhibitors like olaparib may increase the effectiveness of gas plasma. ▪ Gas plasma increased phosphorylation of H2A.X associated with increased DNA damage responses.	^123^
MDA‐MB‐453, MDA‐MB‐231, MCF‐10A	DBD (air) direct treatment	60–120 s, 48 h incubation	▪ Treatment time‐dependent cytotoxicity higher in MCF‐10A than MDA‐MB‐231 and MD‐MB‐453 cells. ▪ NAC and catalase rescued viability only partially in MCF‐10A and not at all in MDA‐MB‐231 cells ▪ Treatment inhibited IL‐6R/pSTAT3 signalling pathway, leading to increased PTEN and decreased AKT phosphorylation. ▪ Gas plasma resistance is mediated by HER2 increase together with ROS scavenging.	^164^
MCF‐7, T‐47D, MCF‐10A	DBD (Ar) direct treatment	30 s each hour 10 times and single 600 s, 24 h incubation	▪ Gas plasma suppressed, depending on treatment condition, BC cells proliferation by down‐regulation of ZNRD1 and its antisense long noncoding RNA ZNRD1‐AS1 expression ▪ Modified the methylation status of CpG sites ▪ Inhibited growth rate and colony formation in ZNRD1 and ZNRD‐AS1 transfected BC cells	^165^
MCF‐7, MDA‐MB‐231, MCF‐10A, MCF‐12A	DBD (Ar) direct treatment	30 s each hour 10 times and single 600 s, 24 h incubation	▪ Increased intracellular reactive oxygen species (ROS) ▪ Induced apoptosis preferentially in BC cells ▪ Changed methylation status in some of the CpG islands in BC cells (DNAJC8, POTED, and EIF1YA) and estrogen receptor‐positive BC cells (ESFR1, PRR7, CD86, DHRS7B, FDX1, CREB3, BCL‐2, and BDNF)	^166^
MDA‐MB‐231	Plasma jet (He) direct treatment	60–300 s, 48 h incubation	▪ Viability decreased in a treatment time‐and input power‐dependent manner ▪ Significant temperature increase in the gas plasma‐treated in vitro cultures	^124^
MCF‐7, BT‐474, MDA‐MB‐231, SK‐BR‐3	Plasma jet (He) direct treatment	120–360 s, 6 h–48 h incubation	▪ Reduced proliferation (Ki‐67) and increased apoptosis and cell cycle arrest in a caspase 3 and 7 and treatment time‐dependent manner ▪ Toxicity varied between different BC cell lines ▪ DNA damage response induced (ATF3, EGR1, ID2) ▪ Histone RNA oxidation proposed to mediate gas plasma toxicity ▪ DNA damage is not the primary mode of BC cell death by gas plasma exposure	^125^
AMj13	DBD (air) direct treatment	5–15 s, 72 h incubation	▪ Gas plasma reduced viability and colony formation in long‐term observation	^167^
MDA‐MB‐231	Plasma jet (Ar) direct treatment	5–25 s, 24 h incubation	▪ Gas plasma reduced BC cell viability in an exposure time‐and input power‐dependent manner	^138^
MDA‐MB‐231	Plasma jet (Ar) direct treatment	5–30 s, 24 h incubation	▪ Gas plasma in combination with a static magnetic field (SMF) and vitamin C decreased cell viability and migration, while SMF alone had no effect	^139^
MCF‐7	Plasma jet (He, He/O_2_, or Ar/O_2_) direct treatment	5–30 s, 48 h incubation	▪ ROS derived from gas plasma treatment increased apoptosis in BC cells ▪ He/O_2_ plasma showed the highest toxicity	^126^
MDA‐MB‐231	Plasma jet (He) direct treatment	5–300 s, 144 h incubation	▪ Exposure time‐dependent toxic effects ▪ Different discharge modes of gas plasma have different anti‐proliferation effect. ▪ Gas plasma toxicity was different in a dose‐dependent‐ manner, and apoptotic cells showed regular DNA fragmentation behaviour	^127^
4T1	Plasma jet (He) direct treatment	1 min, 24 h and 36—38 h incubation	▪ Decrease of metastatic behavior ▪ Augmented apoptosis ▪ Delayed DNA fragmentation in sub G1 phase compared to drug‐controls	^128^
SK‐BR‐3, HaCaT	Plasma jet (N_2_ or N_2_/H_2_O) direct treatment	1–5 min, 48 h (SK‐BR‐3) and 72 h (HaCaT) incubation	▪ Humidified gas plasma exposure led to higher ROS production, cellular oxidation and oxidative stress, and caspase 8‐dependent cell death mediated by p38‐MAPK phosphorylation and ERK inhibition together with PARP‐1 cleavage. ▪ ATM and p53 DNA damage response activated ▪ HaCaT cells are overall less affected	^147^
MDA‐MB‐231, MCF‐10A	Plasma jet (Ar) direct treatment	30 s, 6 h incubation	▪ Up‐regulation of the chemotherapy cationic transporter SLC22A16 gene expression in MDA‐MB‐231 but not MCF‐10A	^72^
MCF‐7, MCF‐10A	Plasma jet (He or He/O_2_) direct treatment	30–300 s, 24 h incubation	▪ Gas plasma selectively induced toxicity on BC cells with negligible effect on low malignant MCF‐10A cells ▪ Gas plasma increased BC cell death through activation of caspases 3 and 7	^129^
MCF‐7	Plasma jet (Ar) direct treatment	30 s each hour 10 times, 24 h incubation	▪ Gas plasma inhibited BC cell growth and recovered drug sensitivity of Tx‐resistant MCF‐7 (MCF‐7/TxR) ▪ Gas plasma‐induced cell sensitivity to drug (Paclitaxel) not related to changes in drug uptake but modification of oncogene and tumour suppressor gene expression (KIF13B, CEACAM1, GOLM1, TLE4, PHKA1, DAGLA) ▪ Gas plasma‐induced up‐regulation of tumour suppressor DAGLA and down‐regulation of tumourigenic CEACAM ▪ Gas plasma decreased drug resistance and toxicity by altering colony formation and tumourigenesis in siDAGLA and siCEACAM transfected BC cells	^140^
MCF‐7	Plasma jet (Ar) direct treatment	30 s each hour 10 times, 2–6 days incubation	▪ Increased toxicity and reduced tumourigenesis in drug (Tamoxifen) resistant BC cells ▪ Gas plasma re‐sensitized BC cells to Tamoxifen by reversing expression of BAG1, CD24 and HDAC4 genes ▪ Decreased colony formation, and tumourigenesis in siMX1 and HOXC6 ORF transfected cells ▪ Altered drug resistance status in BC cells by modification of XRCC, SOX9 and SULT1A1 expression ▪ ROS scavenger inhibited the gas plasma effects	^141^
MDA‐MB‐231, MSC	Plasma jet (He) direct treatment	30–120 s, 24 h incubation	▪ BC cell viability and invasion were inhibited ▪ Non‐malignant MSC cells were less affected	^130^
MDA‐MB‐231, MSC, MCF‐7	Plasma jet (air) direct and nanoparticle treatment	60–90 s, 24h incubation	▪ Increased nanoparticles uptake in BC cells ▪ Higher permeabilization and toxicity when combined with drug‐loaded nanoparticles ▪ Gas plasma down‐regulated MTDH, MMP2, MMP9 and VEGF as indicators of cancer progression	^191^
MCF‐7, HF	Plasma jet (He/O_2_) direct treatment	15–45 s, 24 h incubation	▪ Gas plasma combined with iron NPs changed morphology and activated programmed cell death through BAX/BCL‐2 but not β2‐microglobulin ▪ Less damage in non‐malignant HF cells	^134^
MDA‐MD‐231, MCF‐7	DBD (Ar) direct treatment	30 s each hour 10 times; 100 s and 600 s six times per day; up to 6 days incubation	▪ Tumour suppressor and antitumour properties by increased ABCA1, PTEN, HBP1 and GJA1 expression in miR‐19 transfected and non‐transfected BC cells ▪ Modified methylation status of promoter CPG sites ▪ Decreased cell colonies and proliferation (by increasing ABCA1, PTEN, HBP1, and GJA1 genes expression) in miR‐19 transfected cells ▪ ROS scavenger suppressed gas plasma effects on miR‐19a cells and its target genes	^189^
4T1	DBD (air) direct treatment	10–40 s, 24 h incubation	▪ Treatment time‐dependent ROS formation and toxicity ▪ Activation of ICD pathways (calreticulin ▪ Treated BC cells activated DCs (CD80 and CD86)	^196^
MDA‐MD‐231	Plasma jet (He)‐treated liquid (indirect) and direct treatment	1 min (direct), 2–10 min (indirect), 24 h incubation	▪ Treatment time‐dependent extracellular and intracellular H_2_O_2_ increase for direct treatment while indirect treatment only increased extracellular H_2_O_2_ ▪ Direct gas plasma treatment shows higher toxicity than indirect treatment	^160^
MDA‐MD‐231, MCF‐7	Plasma jet (He) direct treatment	3 and 5 min, 24 h and 48 h incubation	▪ Mitochondria oxidation and metabolic activity decline in 2D BC cells and 3D BC spheroids ▪ DAMPs and ICD‐associated molecules (HSP70, HSP90, calreticulin, PD‐L1, MHC‐I, ATP, IFN‐α2, IFNγ, IL‐6) observed in treated cells	^131^
MDA‐MB‐231, MDA‐MB‐468, MCF‐7, MCF‐10A	Plasma jet (He)‐treated liquid (indirect) and direct treatment	10–50 s (direct), 1–5 min (indirect), 24 h incubation	▪ Indirect exposure in TNBC cells was dose‐dependent ▪ Apoptosis and migration were different in treated TNBC compared to non‐malignant and non‐TN BC cells	^150^
MDA‐MB‐231, human fibroblasts	Plasma jet (He or He/O_2_)‐treated liquid (indirect) and direct treatment	1–5 min, 0 h and 48 h incubation	▪ Direct and indirect exposure selectively decreased BC cell viability due to an increase of ROS and programmed cell death through BAX, BCL‐2, caspase 8 and caspase 3 pathways	^132^
MCF7, HCC1806	Plasma jet (air)‐treated liquid (indirect) and direct treatment	15–120 s (direct), 60–120 s (indirect), 24 h incubation	▪ Reduced BC cell protein content and metabolic activity in a treatment time‐dependent manner	^151^
MCF‐7, MDA‐MB‐231, MCF‐10A	DBD (He)‐treated cell culture medium (indirect)	45–240 s, 24 h and 48 h incubation	▪ Long but not short treatment times affected mitochondrial activity and BC cell growth and migration ▪ Presence of FBS supposedly increased OH radical and H_2_O_2_ production ▪ Metastatic BC cells are more sensitive	^169^
MDA‐MB‐231, primary murine fibroblasts	DBD (Air)‐treated DI‐water (indirect)	18 min, 24 h incubation	▪ More toxic in BC cells over fibroblasts ▪ Less toxicity the longer time difference from gas plasma‐treated liquid generation to application ▪ Induced cell death was not related to higher acidity	^170^
SUM149PT, SUM159PT, MDA‐MB‐231, MDA‐MB‐436, MCF‐7, SK‐BR‐3, MCF‐10A	Plasma jet (He)‐treated liquid (indirect)	1–2 min, 24 h incubation	▪ Toxicity depends on cell density, treatment time, gas flow rate, plasma device output voltage, well size, and plasma effluent distance to the liquid ▪ Gas plasma induces higher toxicity in triple‐negative (TN) cancer cells compared to non‐TN cells may result in more AQPs on their cell surface ▪ Gas plasma is a promising treatment method for cancer stem cells and can inhibit metastasis	^152^
MCF‐7, MDA‐MB‐231, MCF‐10A	Plasma jet (Air)‐treated liquid (indirect)	30–120 s, every week for 10 week	▪ Increased oxidative stress and cell cycle arrest, and decreased proliferation ▪ Programmed cell death through activation of BAX and PUMA, cytochrome C release, and caspase activation ▪ Elevated H2A.X phosphorylation and p53 activation	^153^
MDA‐MD‐231, MCF‐7	Plasma jet (He)‐treated liquid (indirect)	.5–2 min, 24 h incubation	▪ Exposure time, well plate size, cell density, volume of media and distance of gas plasma with treated surface affect relative ROS levels and cytotoxicity toxicity ▪ H_2_O_2_ levels and RNS affected by host cell amino acids, especially cysteine and tryptophane, and weakened intracellular antioxidant system ▪ Absorption and elimination of gas plasma‐produced ROS varied among different BC cell lines	^154^
MCF‐7, MDA‐MB‐468, MDA‐MB‐231	Plasma jet (Ar)‐treated cell culture medium (indirect)	10 min, 24 h incubation	▪ Mesenchymal BC cell lines showed more susceptibility than epithelial counterparts ▪ Sensitivity of luminal subtypes lower than basal subtypes ▪ ROS levels were dramatically higher in mesenchymal compared to epithelial breast cancer cells ▪ Cells with a higher level of epithelial‐mesenchymal transition (EMT) score are more sensitive, as evident by a weighted analysis of 76 EMT‐related genes ▪ 10% fetal bovine serum in gas plasma‐treated media decreased cytotoxic treatment efficacy BC cells from being damaged after gas plasma‐activated media ▪ Exposure modified epithelial and mesenchymal gene expression correlating with metastasis and invasion inhibition	^155^
MDA‐MD‐231	Plasma jet (He)‐treated DI‐water (indirect)	5–30 min, 24 h and 48 h incubation	▪ ROS produced by gas plasma in DI water decreased metabolic activity of BC cells with increasing exposure time	^156^
MDA‐MD‐231	Plasma jet (Ar, N_2_, He)‐treated DI‐water (indirect)	Not stated treatment time, 24 h and 48 h incubation	▪ Ar‐treated liquids produced more ROS and were more cytotoxic than N_2_‐ and He‐treated liquids ▪ Effects in gas plasma‐treated cell culture media were greater than in non‐media liquids	^218^
MDA‐MB‐231	Plasma jet (Ar)‐treated liquid (indirect)	180 s, 24 h incubation	▪ Gas plasma alone and combined with FK866, a Nicotinamide phosphoribosyltransferase (NAMPT) inhibitor, increased intracellular ROS and cytotoxicity ▪ PARP‐1 activation and cell death associated with energy constraints by NAD+ and ATP level depletion ▪ ∆Ψm disruption and mitochondrial dysfunction ▪ Weakened antioxidant defense system (reduction of GSH and NADPH) ▪ siRNA‐mediated NAMPT increased plasma cytotoxicity	^157^
MDA‐MB‐231, MCF‐7	Plasma jet (Ar) direct treatment	20 s, 24h incubation	▪ Non‐cytotoxic treatment condition ▪ Increased expression of the immune checkpoints and inflammation‐related surface molecules CD40 and CD112, as well as CD273 and Gal‐9 in MDA‐MB‐231 and CD271 in MCF‐7 cells	^142^
MDA‐MB‐231, MCF‐7	Plasma jet (Ar) direct treatment	60–180 s, 6 h and 24 h incubation	▪ Increased intracellular ROS ▪ Cleaved PARP‐1 and HSP90β and PKD2 degradation, and affect enhancement via HSP90 inhibitor PU‐H71	^143^
MDA‐MB‐231, MCF‐7	Plasma jet (Ar) direct treatment	30–120 s, 20 h incubation	▪ In a 36 cancer cell line comparison, MCF‐7 and MDA‐MB‐231 were of high and low resistance, respectively, to gas plasma treatment ▪ Both cell lines showed modestly enhanced NOX3 and AQP1 expression that had a good correlation with gas plasma‐induced cytotoxicity	^144^
MDA‐MB‐231, MCF‐7	Plasma jet (Ar)‐treated cell culture medium (indirect)	60 min treatment of 50 ml, exposure to 100 μl	▪ Modest cytotoxic effects ▪ No major change in the expression of 26 redox‐related transcripts (peroxiredoxins, glutaredoxins, etc.) except for HMOX1, especially in MCF7 ▪ No major effect on ICD and immunology‐related molecules (CD47, CRT, HLA‐ABC, HSP70, HSP90) in modest treatment conditions	^158^
MDA‐MB‐231	Plasma jet (Ar) direct treatment	30–60 s, 6h and 24 h incubation	▪ Relatively low resistance to gas plasma‐induced toxicity when compared to 10 other cell lines ▪ Exceptionally high oxidized GSH after exposure	^145^
MDA‐MB‐231, MCF‐7	Plasma jet (He) direct treatment	120–300 s, 6 h–48 h incubation	▪ Treatment time‐dependent generation of H_2_O_2_, NO_2_^–^, and HOCl with modest pH increase in culture medium ▪ Treatment time‐dependent cytotoxicity and decline in metabolic activity in 2D cultures and 3D tumour spheroids ▪ At 24h and 48h, increased surface expression of CRT, HSP70, HSP90, MHC I, and PD‐L1 in both cell lines ▪ LC3 (autophagy) increase in MCF‐7, and elevated ATP and HSP70 secretion in both cell lines	^131^
MDA‐MB‐231, T‐47D, SK‐BR‐3, MCF‐7, BT‐474, HS578T	Plasma jet (He) direct treatment	300 s, 3–24 h incubation	▪ Analysis of 48 apoptosis and 35 oxidative stress genes after plasma treatment ▪ BCL2A1 increased only in plasma‐treated TNBC lines ▪ Silencing BCL2A1 augmented plasma‐induced cytotoxicity in breast cancer cells ▪ BCL2A1 was described before to contribute to malignancy and chemoresistance	^133^
MCF‐7	Plasma jet (Ar)‐treated culture medium (indirect)	60–240 s, 48 h incubation	▪ Plasma‐treated medium combined with doxorubicin treatment ▪ Intracellular ROS generation and metabolic activity decline ▪ Combination effect observed	^159^
MDA‐MB‐231	DBD (air) direct treatment	60–120 s, 24 h incubation	▪ Increased ROS and decreased viability in plasma treatment time‐dependent manner ▪ Correlated with decreased HIF‐1α and VGEF expression	^168^
BT‐474	Plasma jet (Ar/O2) direct treatment	60–150 s zz, 24 ‐ 72 h incubation	▪ Nearly 90% reduced cell viability already with 60 s and at 24 h, with not much increase of cell death with longer gas plasma treatment or incubation times	^146^

**TABLE 4:** In vivo and ex vivo studies and findings in gas plasma breast cancer treatment. BC = breast cancer

Model	Gas plasma device	Exposure modality	Gas plasma treatment effects and mechanisms of action in breast cancer cells	Ref.
AN3 cells, female Swiss albino mice (6–8 weeks), xenograft	DBD (air) direct treatment	20–50 s, three times, every 48 h, 21 days follow‐up	▪ Decreased tumour growth ▪ Modestly increased survival ▪ Decreased body weight gain	^163^ ^]^
**4T1 cells, female BALB/c mice (4–‐6 weeks), syngeneic**	DBD (He) direct treatment	180 s every 48 h, 25 days follow‐up	▪ Reduced tumour volume and enhanced survival ▪ Increased BC tumour apoptosis and altered BAX and BCl‐2 as well as p53 expression	^128^
**MDA‐MB‐231 or MCF‐7 cells, in female BALB/c (4–6 weeks), xenograft**	Plasma jet (He)‐treated cell culture medium for injection	100 μl 15‐min plasma‐treated medium, daily 29 days	▪ No effect in MCF‐7 tumour xenografts ▪ 80% weight and tumour volume reduction in MDA‐MB‐231 xenografts	^150^
**4T1 cells, female BALB/c mice (4–5 weeks), syngeneic**	DBD (air) direct treatment	1–4 min tumour wound plasma treatment immediately after surgery, 60 days follow‐up	▪ Decreased relapse of tumour growth from surgically removed tumour wounds with gas plasma treatment, and enhanced survival ▪ The effect was treatment‐time dependent ▪ Gas plasma‐induced ICD locally and enhanced DC maturation (CD80, CD86)	^196^
**4T1 cells, female BALB/c mice (6–7 weeks), syngeneic**	Plasma jet (He) direct treatment	5 min, daily, for 21 days	Tumour growth and volume reduction True abscopal effect with tumour reduction on the plasma‐treated flank and in parallel reduced growth of an untreated tumour of the opposite flank in the same animal Plasma‐induced ICD locally, enhanced apoptosis and CRT expression, increased leukocyte infiltration (DC, CD4^+^ and CD8^+^ T‐cells) in both the untreated and gas plasma‐treated tumour flank	^131^
**Breast cancer patient‐derived tumour tissue minced and gas plasma‐treated ex vivo**	Plasma jet (He) direct treatment and plasma‐treated medium	5 min, two times on two consecutive days, 24 h incubation	▪ Gas plasma elevated tumour cell death in microtissues by activation of caspase 3 and reduced microsatellite growth in a 3D model system ▪ Reduced migratory activity of Tumour cells in microtissues ▪ Gas plasma exposure modified the release of several inflammatory agents in the TME, such as IL‐6, IL‐8, IL‐18, IL‐33, IL‐17A, IFN‐α2 and MCP1	^120^

### 2.1 Cytotoxicity and ROS production

An extensive array of plasma devices and feed gas settings was used across different studies to investigate the toxicity in breast cancer cell lines in vitro. Cytotoxic effects were used in the direct treatment regimen with plasma jets driven with helium (He),^121^, ^122^, ^123^, ^124^, ^125^, ^126^, ^127^, ^128^, ^129^, ^130^, ^131^, ^132^, ^133^ which was the setting mostly applied in the literature. In some studies, the direct He plasma treatment was compared with Helium‐oxygen (He/O_2_) plasma treatment,^123^, ^126^, ^129^, ^132^, ^134^ and generally, the He/O_2_ conditions were found to be more toxic than the He condition. This is because oxygen addition, especially to helium, increases the presence of atomic and singlet delta oxygen in the plasma gas phase.^135^ If not quenched by molecular oxygen to generate ozone, oxygen addition can lead to hypochlorous acid formation, regardless of whether helium or argon (Ar)^136^ is used as carrier gas. Also other cell lines were found before to be more sensitive to He/O_2_ than He direct plasma jet treatment.^137^ Direct Ar plasma jet treatment of breast cancer cell lines was also reported in various studies.^72^, ^138^, ^139^, ^140^, ^141^, ^142^, ^143^, ^144^, ^145^, ^146^ With regard to plasma jets, one study compared nitrogen (N_2_) against humid nitrogen (N_2_/H_2_O) and found the latter to be significantly more toxic than the former.^147^ This can be explained based on earlier findings identifying a critical role of humidity in the feed but not in ambient air. Specifically, humidity in the feed gas increases the production of H_2_O_2_ and one of its precursors, OH radicals, directly in the plasma gas phase.^148^ Accordingly, treated liquids show several‐fold increased ROS production and much higher cytotoxic effects if humidified feed gas is used.^149^ As stated above, the ROS produced by plasmas in liquids can also be used in the so‐called indirect plasma treatment, where plasma‐treated liquids are added to tumour cells to perform cytotoxic action.^88^ Several studies used such an approach using plasma jets operated in different feed gas settings and utilizing different types of treated liquids to successfully induce cytotoxicity in breast cancer cell lines in vitro.^132^, ^150^, ^151^, ^152^, ^153^, ^154^, ^155^, ^156^, ^157^, ^158^, ^159^ On average, indirect plasma treatment was comparable to direct treatment effects in those studies where a side‐by‐side comparison was performed. However, one study found indirect treatment to only increase extracellular but not intracellular ROS and induced remarkably less cytotoxic, than direct treatment.^160^ This can be explained by the cell culture medium used to perform the plasma treatment. Earlier reports already pointed to a pivotal role of the composition of liquid to be plasma‐treated on the latter effects in the cells exposed to these liquids.^161^ Specifically, some media types are enriched with greater levels of antioxidants such as pyruvate, which scavenge ROS and thereby reduce the plasma treatment effect. On a relative scale, the scavenging role of fetal bovine serum often supplemented to cell culture media to provide growth factor stimulation is less pronounced, at least in terms of eukaryotic plasma treatment in vitro.^149^ Hence, plasma‐treated medium containing high levels of ROS scavengers quickly lose their cytotoxic effects after being generated (within minutes to hours, depending on storage), while during direct plasma treatment of cells immersed in those media, the scavenging kinetic might be too slow to shield all cells from the effects of plasma‐derived ROS. In general, the generation of plasma‐treated cell culture media is an academic endeavor rather than of clinical significance. Similar to the need for medical devices’ approval in order to be used in clinical applications, only approved types of liquids can be used in patients. Such liquids include, for instance, .9% sodium chloride and Ringer's lactate, both showing favorable effects as plasma‐treated liquids in a broad comparison on the utilization of plasma‐treated liquids for anticancer effects.^162^ In addition, there are many studies on direct^128^, ^163^, ^164^, ^165^, ^166^, ^167^, ^168^ and a few on indirect^169^, ^170^ DBD plasma breast cancer treatment. Overall, results did not differ profoundly in terms of a principle induction of cytotoxic effects as found in the plasma jet studies. The DBDs were operated mostly in ambient air or a feed gas, such as argon, was used. Unfortunately, none of the studies using jet or DBD plasma‐treated liquids (indirect approach) used a type of liquid approved for clinical use. This gap might be filled by future studies.

### 2.2 Mode of action and selectivity

**FIGURE 4:**
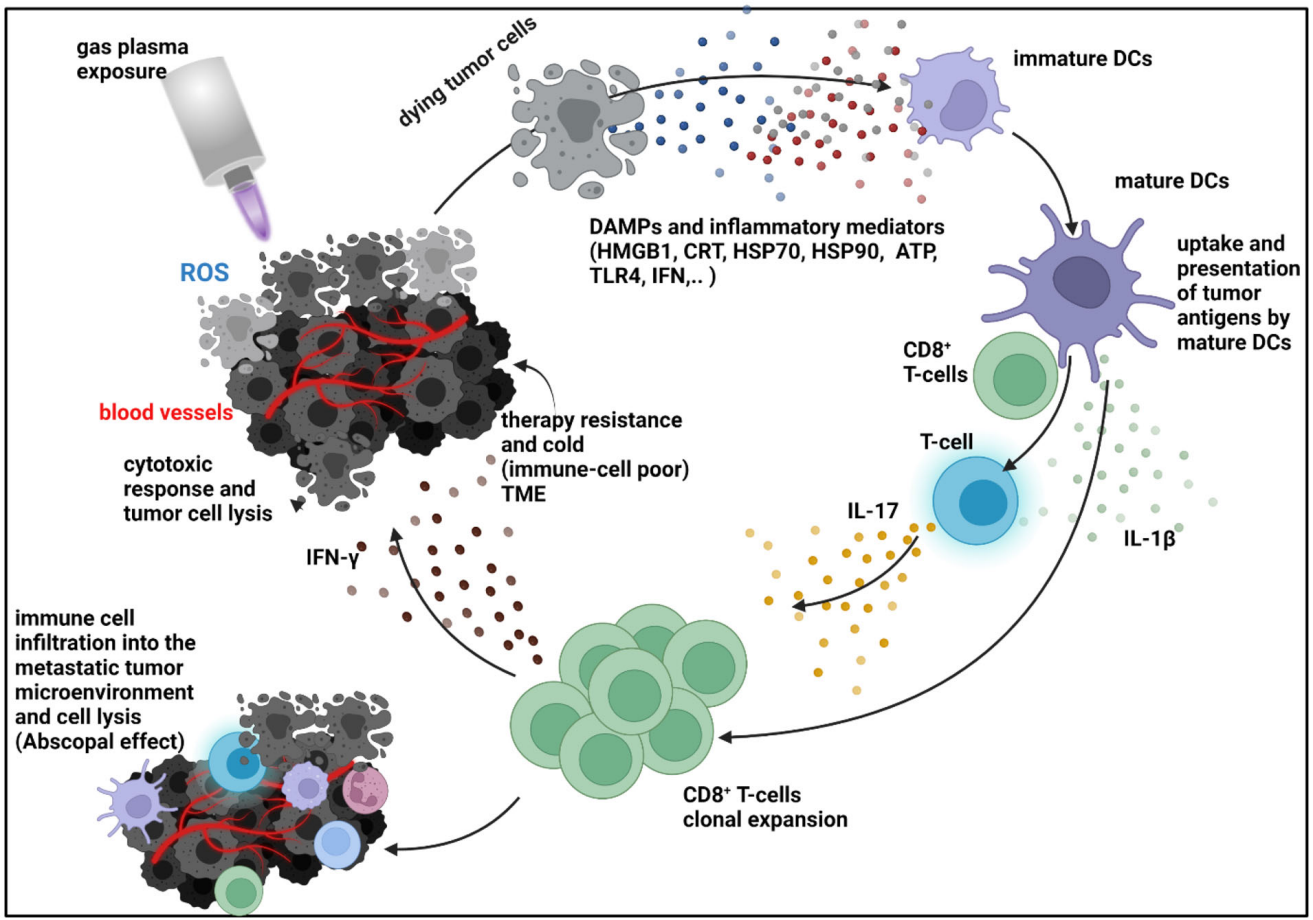
The cancer‐immunity cycle involves immunogenic cell death (ICD) and release of damage‐associated molecular patterns (DAMPs), spurring dendritic cell (DC) maturation and cognate antigen effector T‐cell activation and clonal expansion contributing to anticancer immunity. Gas plasma exposure is hypothesized to kick‐start this cycle, as shown in two published in vivo studies.^131^, ^196^ The image is adapted based on established ICD concepts.^116^, ^217^

Since gas plasmas produce sufficient amounts of ROS, it is understood that gas plasma‐mediated (breast) cancer cell death is in line with many findings in redox biology and ROS‐dependent initiation of cell death.^37^ Most of these pathways lead to the initiation of apoptosis (Figure 4). However, it should be mentioned that the involvement of other forms of regulated cell death has been mentioned in tumour cells subsequent to gas plasma exposure, such as autophagy and lysosomal cell death,^171^, ^172^, ^173^, ^174^, ^175^ ferroptosis^176^, ^177^, ^178^, ^179^ and necroptosis.^172^

In contrast to other physical modalities, such as ionizing and UV radiation, where ROS are generated intracellularly and extracellularly, gas plasma produces majorly extracellular ROS because this modality is void of extensive radiation. Therefore, extracellular biomolecules and cell membranes are the first targets of these ROS. Simulation studies on gas plasma‐treated membranes predicted the possibility of short‐term ROS‐induced pore formation, which may help surplus extracellular ROS to enter tumour cells.^180^ In parallel, lipid peroxidation was predicted to occur, likely via hydroxyl radical formation.^181^ Albeit several details on gas plasma‐induced lipid oxidation remain elusive, it is assumed that lipid head group oxidation takes place prior to tale oxidation.^182^ In addition, cholesterol‐poor cell membranes, as frequently observed in tumour cells, were predicted to be more sensitive to gas plasma‐mediated damage compared to cholesterol‐rich counterparts, as suggested by computer simulations.^183^ Recently, a systemic comparison between different tumour cell lines underlined this notion.^144^ Another frequently described class of membrane transporters critical in breast cancer progression is aquaporins.^184^ The transmembrane channels are known to transport not only water but also ROS, such as hydrogen peroxide, across cell membranes. Accordingly, a role of aquaporins in gas plasma‐induced cancer cell death has been assumed in several works.^152^, ^185^, ^186^ However, a study across 36 cancer cell lines, including several of breast origin, did not support this view.^144^

Once gas plasma‐derived ROS have entered the cell and/or induced sufficient oxidation of, for example, cell membranes, the tumour cells sense the oxidative stress and respond via specific signalling and altered transcriptional and translational profiles. Several in vitro works addressed the mechanisms of gas plasma‐induced cytotoxicity in breast cancer cells. One report indicated that gas plasma could inhibit the growth and proliferation of breast cancer cells by reducing STAT3.^164^ This was linked to decreased IL‐6R expression and Akt phosphorylation, while PTEN was enhanced. STAT3 signalling is associated with breast cancer malignancy by driving progression, proliferation, metastasis and chemoresistance. Therefore, it is investigated as a diagnostic and therapeutic target in breast cancer therapy.^187^ Another study found increased phosphorylation of p38 MAPK and decreased ERK phosphorylation following gas plasma exposure in breast cancer cells,^147^ suggesting cell death initiation. This was accompanied by elevated p53 and caspase 8 expression levels, and other reports supported this by finding increased caspases 3, 7, 8 and 9 levels in gas plasma‐treated breast cancer cells.^125^, ^163^ BAX and PUMA activation, as well as mitochondrial oxidation and cytochrome C release, were also found.^131^, ^132^, ^153^ Apoptosis induction via p53 phosphorylation and increased Bax/Bcl2 ratios were also reported in gas plasma‐treated breast cancer tumours in vivo in mice.^128^ Elevated apoptoitic events increased breast cancer cell expression of ATF3, a molecule implicated in DNA damage responses, which was also mentioned in this study. It is assumed that those findings, along with several studies that reported elevated phosphorylation levels of the DNA‐damage indicator histone 2A.X,^123^, ^127^, ^153^ are secondary events and not the action of primary gas plasma‐generated ROS reaching the DNA directly.^74^ Gas plasma exposure also increased the expression of HMOX1 in MDA‐MB‐231 and MCF‐7, and glutaredoxin 1 and peroxiredoxins 1 in the latter.^158^ These proteins are involved in antioxidant defenses and DNA‐damage responses, and the data align with findings of exceptionally high oxidized GSH (GSSG) levels in gas plasma‐treated breast cancer cells.^145^ This is supported by data on cell death abrogation in gas plasma‐treated breast cancer cells by prior addition of the amino acids cysteine and tryptophane,^154^ both known to scavenge ROS and support cellular antioxidant defense.

Extracellular matrix broken‐down by matrix metalloproteinases after epithelial‐mesenchymal transition, together with decreased integrin and cadherin expression, causes cancer cells to enter the bloodstream or lymph vessels, promoting metastasis.^188^ Gas plasma treatment of breast cancer patient‐derived microspheres in collagen was accompanied by elevated E‐cadherin expression.^120^ According, decreased migration was reported for gas plasma‐treated breast cancer cells in transwell migration and matrigel invasion as well as 2D time‐lapse video microscopy cell motility assays.^130^ This is supported by findings on reduced TRIM31 expression in breast cancer cells following gas plasma exposure,^165^ a molecule involved in tumour progression and metastasis. The molecular basis of these changes is not understood yet. A genome‐wide epigenetic study on gas plasma‐induced changes in CpG methylation sites identified in an IPA analysis genes associated with cell‐to‐cell signalling as top category,^166^ suggesting an involvement of junctional and adhesion kinase signalling processes. As a more basic mechanism underlining gas plasma‐derived ROS mechanisms, a recent report suggested oxoguanine (8‐oxoG) modifications of mRNA to be critical, hypothesizing that these messengers would be removed and specifically affect S‐phase cell cycle progression,^125^ albeit is it unclear how such specificity would be achieved. Recent studies also implied regulation on the micro RNA level, with miR‐19a having a particularly tumour‐promoting role in breast cancer cells that were attenuated by gas plasma treatment.^189^  The potential of gas plasma technology for targeting breast cancer Sander Bekeschus and Fariba Saadati equally contributed as first authors.

### 2.3 Plasma combination treatment and drug resistance

Several studies combined gas plasma exposure with standard or experimental anti‐breast cancer agents in vitro. In an extensive comparison study using 14 different breast cancer cell lines with different molecular profiles (luminal, basal A, basal B), gas plasma was combined with radiotherapy.^123^ Generally, all cell lines were sensitive to both types of treatments, albeit on a different exposure scale. However, what was unexpected was a highly significant correlation between both treatments, that is, cells sensitive to gas plasma were also sensitive to radiotherapy and vice versa. This indicates that radiotherapy—at least in vitro—is considerably acting via ROS generation, inducing oxidative stress and cell death. The second intriguing finding of the study was that gas plasma and radiotherapy synergistically combined when exposed together in the seven breast cancer cell lines investigated. The third notion was that gas plasma treatment combined with the PARP‐inhibitor olaparib to elevate toxicity, especially in TNBC. Albeit, underlining data in animal models are missing so far, the many combinations and cell lines employed in this study outline the potential gas plasma treatment might with established breast cancer therapies.^123^ Another conventional breast cancer chemotherapy is paclitaxel. Gas plasma exposure significantly reversed paclitaxel resistance in MCF‐7 cells.^140^ Transcriptomic analysis revealed several potential candidates mechanistically linked to this finding, and CEACAM1 and DAGLA were found to regulate sensitivity restoration to the drugs. The two proteins had so far not been ascribed a role in breast cancer drug resistance. Another report investigated the effects of gas plasma‐derived ROS in tamoxifen drug‐resistant MCF‐7 cells.^141^ The treatment reduced tamoxifen sensitivity in 50% of the cells, and following a gene expression profiling, siRNA‐mediated knockdown provided evidence of MX1 and HOXC6 playing a role in drug sensitivity restoration via oxidative stress. The former is known to act in cancer mitosis, while the latter is associated with the mammary gland and described in multidrug‐resistant cancer cells. Experimental drugs and approaches have also been combined with gas plasma exposure of breast cancer cells in vitro. For instance, with ascorbate, a substance frequently debated to be beneficial also in breast cancer patients,^190^ additive cytotoxicity with gas plasma exposure could not be identified in human breast cancer cells in vitro.^139^ Another study reported a modest combination of gas plasma with gold nanoparticles in two breast cancer cell lines falsely claimed to be synergistic.^191^ Gas plasma treatment alone and combined with FK866, a nicotinamide phosphoribosyltransferase inhibitor, increased intracellular ROS and cytotoxicity via PARP‐1 activation, ∆Ψm disruption and mitochondrial dysfunction and weakened antioxidant defense system (reduction of GSH and NADPH), leading to energy crisis and ATP depletion in MDA‐MB‐231 breast cancer cells.^157^ Gas plasma exposure also potently combined with PU‐H71, an HSP90 inhibitor currently in clinical trials, via enhanced degradation of PKD2 and HSP90β cleavage in breast cancer cells.^143^ However, translational models are lacking to validate any therapeutic implication of the findings, but the mentioned studies nevertheless indicate a role of oxidative stress in combination treatments of breast cancer cells.

### 2.4 Plasma‐induced ICD in breast cancer

Several physical treatment modalities have already been observed to induce ICD.^192^ The idea is to promote antitumour immunity by enhancing the quality and quantity of T‐cells targeting tumour cells. The concept has been extended to gas plasma cancer treatment by framing and experimentation (in a syngeneic colon cancer mouse model) a few years ago,^193^, ^194^ with the gas plasma process functioning as a *kick‐starter* to set the cancer‐immunity‐cycle in motion (Figure 4). It should be noted that direct gas plasma‐mediated tumour debulking is only a secondary outcome in this process, as there are other methods more suitable to provide such effect. The primary goal is localized ICD induction, sometimes called in situ vaccination.^195^ One in vitro report screened the expression of 18 immune‐related surface markers after low‐toxicity gas plasma treatment and found an increased expression of CD40 and CD112, as well as CD273 and Gal‐9 in MDA‐MB‐231 and CD271 in MCF‐7 cells, indicative of oxidative stress translating to differential immuno‐suppressive signatures.^142^ Of the three studies investigating the anticancer efficacy of gas plasma treatment in syngeneic breast cancer mouse models, two deliberately investigated immune effects. In the first study, a helium‐driven plasma jet (kINPen) treatment induced hallmarks of ICD in MCF‐7 and MDA‐MB‐231 human breast cancer cells in vitro, such as increased externalization of CRT, HSP70 and HSP90 as well as elevated ATP release.^131^ Enhanced CRT externalization was also observed in gas plasma‐exposed 3D tumour spheroids. In vivo, live 4T1 cells were injected into both flanks of mice to grow tumours. Only one site was gas plasma‐treated, leading to growth reduction on this site. Strikingly, tumour growth also significantly declined on the untreated contra‐lateral site, indicating gas plasma inducing an abscopal effect in vivo. TME analysis revealed enhanced apoptosis in both tumours of plasma‐treated mice, along with elevated numbers of intratumoural T‐cells and DCs.^131^ The second study also observed hallmarks of ICD induction in gas plasma‐treated 4T1 cells in vitro, such as CRT exposure and enhanced expression of the DC maturation markers CD80 and CD86 in a plasma treatment time‐dependent fashion.^196^ In vivo, tumours were grown to 500mm^2^ before being surgically removed. The surgical margin was exposed to gas plasma or left untreated, and the tumour relapse growth and animal survival were significantly improved in gas plasma‐treated animals. In regrowing tumours, gas plasma exposure significantly increased intratumoural T‐cells and their proliferation along with elevated levels of IFN‐γ, TNF‐α, IL‐2, IL‐12 and also immuno‐suppressive IL‐10. In future experiments, it would be interesting to see whether the combination of local gas plasma therapy with systemic checkpoint immunotherapy results in augmented anticancer effects. In syngeneic melanoma models, gas plasma‐inactivated melanoma cells in vitro were injected as vaccine in mice, providing partial protection from subsequent live‐cell injection‐related melanoma growth.^197^, ^198^ Also such gas plasma‐assisted vaccination may be combined with other immunotherapies to test for additive antitumour effects. Last but not least, breast cancer patient‐derived samples were recently investigated for their release of inflammatory mediators after gas plasma exposure and incubation ex vivo.^120^ Besides several significant but small‐scale differences for several cytokines, the largest changes were observed for IL‐6 and IL‐8 (decrease > 100‐fold). Both IL‐6 and IL‐8 are pleiotropic agents described to perform immunosuppressive functions in breast cancer.^199^ Hence, it is possible that gas plasma treatment elevates the immuno‐suppressive profiles of breast cancer cells not immediately succumbing to cell death. This has also been observed with other physical modalities for breast cancer treatment before, such as radiotherapy or hyperthermia.^200^ For those reasons, it is assumed in the field of onco‐immunology that physical modalities may help freeing tumour antigen locally to promote antitumour immunity that a later stage should then be re‐invigorated using immunotherapy, such as checkpoint inhibitors.

## 3 GAS PLASMA TECHNOLOGY FOR ULCERATING BREAST CANCER COMBINATION THERAPY

Several clinical treatment modalities are conceivable using gas plasma technology in breast cancer treatment. First, it should be emphasized that only clinically approved gas plasma devices should be used in patient studies to ensure a safe application and approval based on pre‐existing clinical evidence. For plasma jets, devices that have ideal geometries for treating the uneven topology of human body surfaces and surgical wounds, the only approved device for broad applications on the skin or wounds currently is the kINPen MED.^40^

Intuitively, one would assume that plasma could be used to treat breast cancer below the skin. However, this is not feasible as the plasma‐generated ROS have only limited penetration depths that depend on the tissue in question. Non‐keratinized tissues may show effects between a few dozen to a few hundred micrometers.^98^, ^201^ However, in keratinized skin, plasma effects may not reach much beyond the stratum corneum, as evident in human skin plasma‐exposed ex vivo and murine skin (nude mice) plasma‐exposed in vivo.^97^, ^202^ However, deeper tissue effects of the plasma treatment were observed, for instance, in mice skin plasma‐treated ex vivo using hyperspectral imaging as increased tissue water index and deeper tissue oxygenation were noted in these models.^203^ Blood flow elevation, including hemoglobin index, was also found in intact human skin.^99^, ^100^ The mechanisms of action are unclear. Yet, what remains clear is that subdermal breast cancer will likely not be targetable by superficial plasma treatment.

**FIGURE 5:**
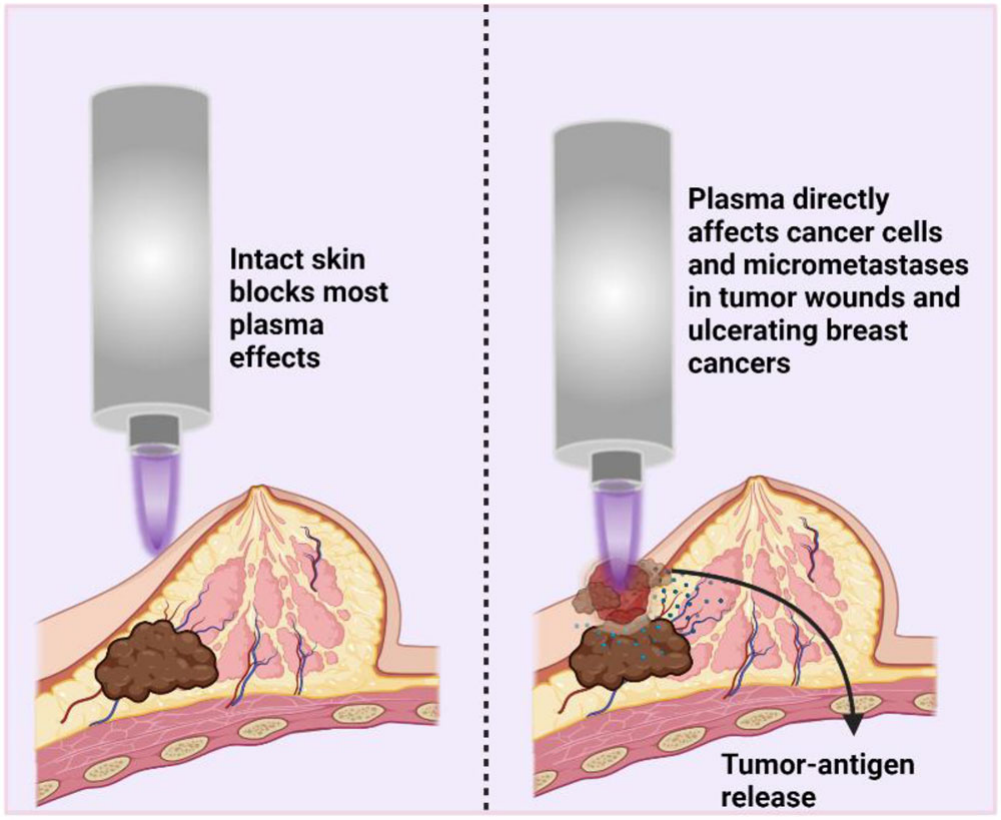
Graphic illustrating the advantage of gas plasma treatment of tumour wounds or ulcerations

In the case of ulcerating breast cancers, however, plasma therapy may be beneficial (Figure 5). This can be deduced from a case report series of patients suffering from advanced head and neck cancer. The palliative cancer patients had ulcerating tumours in the face or neck region, which were heavily colonized with microorganisms, producing a strong odor that negatively impacted the palliation of these end‐stage patients that had failed all standard therapies.^102^ With the original aim of reducing the microbial flora, and hence the odor, more than 20 patients received gas plasma therapy regularly (1–3 times per week) over several weeks to months. A dramatic and unexpected decline of tumour mass was observed in some patients after 1–2 dozen treatment sessions. We had recently hypothesized that the result might have been a combined effect of the direct anticancer action of gas plasma and dead microorganisms acting as in situ adjuvants to boost antitumour immunity.^204^ Eventually, all patients relapsed irrespective of the continuation of plasma therapy and deceased. However, these results provide hope that gas plasma technology might be beneficial in ulcerating and infected breast cancer wounds to simultaneously reduce cancer growth and local infection. When breast cancer ulcerates, they cause various symptoms such as pain, itchiness, unpleasant smell, bleeding, leaking or oozing and infection.^6^ Considering the large number of stage III‐IV breast cancer patients globally, many patients (about 1% of all cases) are prone to developing fungating or infected superficial breast cancer ulcerations.^205^

Another concept of utilizing gas plasma technology is its adjuvant application to surgical breast tumour removal. As emphasized above, recent in vivo studies provided compelling evidence that post‐operative plasma treatment of the tumour resection margins significantly decreased subsequent tumour relapse, probably by reducing tumour micrometastases in the wound margins and promoting antitumour immunity.^196^ Depending on the plasma device to be used, the plasma treatment time might be a reasonable investment for post‐surgical breast cancer wound care, even if recurrence rates would be decreased by this method only by a few percent. This is because gas plasma technology is highly advantageous with regard to its superior safety profile and tolerability.^40^ Clinical plasma treatment is straightforward to implement and apply, and single treatment session costs are negligible before the tumour wound is closed.

An additional concept, which has so far not been explored for breast cancer but for melanoma and colorectal cancer in experimental models, is gas plasma‐assisted tumour vaccination. Using in the laboratory plasma‐inactivated melanoma cells as a vaccine, three independent studies provided evidence of vaccination partially protecting mice from subsequent tumour growth after a second live tumour cell injection.^194^, ^197^, ^198^ In the experiments, plasma treatment did not outperform immunogenic drugs such as the ER‐stress inducer mitoxantrone predicted to elicit ICD,^118^ but possibly, combining plasma treatment with other ICD inducers and adjuvants may improve vaccine‐mediated antitumour protection. In the clinical setting, such an approach may re‐vitalize the long‐standing idea of using autologous tumour material for vaccination purposes by upgrading its immunogenicity using plasma processes. There is no clinical evidence to date to support this concept using gas plasma technology, but autologous tumour lysates and their oxidation using hypochlorous acid were recently shown to induce potent antitumour immunity and improved survival in a cohort of ovarian cancer patients.^206^, ^207^ This promotes the underexplored idea of using ROS as an immune‐enhancer and in autologous anticancer vaccination.^208^

Finally, the idea of post‐surgical plasma treatment of breast cancer wounds and the concept of plasma‐treating ulcerating and infected breast cancer wounds may be combined with other treatment modalities, such as checkpoint immunotherapies. Other physical, local treatment modalities, such as radiotherapy and PDT, have already been explored similarly.^209^ Intriguingly, the in vivo study using plasma treatment to treat the surgical breast tumour margins convincingly showed elevated antitumour immunity, DC activation, and tumour immune infiltrations in the plasma compared to the control group.^196^ Using a syngeneic melanoma model, the same group provided compelling evidence of beneficial combination effects of local plasma tumour treatment with anti‐PD‐1 checkpoint antibody immunotherapy.^210^ Hence, it is conceivable that such an in situ vaccination using gas plasma technology may be worth testing in the clinical setting. Such as approach, similar to the autologous plasma‐upgraded tumour vaccine idea, is especially auspicious since most patients do not die because of the primary tumour but due to metastasis. However, local tumours could be used as biopsy source or in situ vaccination target to help promote existing T‐cell responses (quantity) or support the generation of novel T‐cell clones (quality) adapted to changing mutational landscapes and peptide profiles high‐grade tumours often exhibit.^211^ As similar effects have been ascribed to radiotherapy via release of DAMPs, inducing ICD, and reasoning radio‐immunotherapeutic schemes,^212^, ^213^ it is conceivable that radio‐ and gas plasma therapy may have synergistic, additive or antagonistic effects in local immuno‐stimulation. Our recent combination treatment in vitro using melanoma cells at least suggested a combined immuno‐stimulatory potential.^214^ However, there seems to be unraised potential in combining both technologies in translationally more relevant models, especially since radiotherapy is already clinically used to treat ulcerating breast cancer.^215^ The ultimate goal remains to enhance the efficacy of immunotherapies by supporting antigen release and inflammatory presentation conditions rather than tumour mass debulking, as recently summarized in the case of adjuvant radiotherapy.^216^ In this frame, gas plasma technology may be an additional future tool in the therapists’ toolbox to be added depending on the patient's tumour characteristics to help foster tailored anticancer treatments.

## 4 CONCLUSION

Gas plasma‐derived ROS are a promising adjuvant tool to support existing breast cancer therapies. Although clinical evidence on plasma breast therapy success is absent, promising proof‐of‐concept studies are available for other tumour entities in patients and breast cancer animal models. Auspicious applications involve the plasma's capability to increase breast cancer cells' immunogenicity and use the technology for in situ or ex vivo vaccination approaches. Particularly, patients suffering from ulcerating and infected breasts are expected to benefit from gas plasma therapy.

## CONFLICT OF INTEREST

The authors have no conflict of interest to declare.
